# Differentiation between pine woods according to species and growing location using FTIR-ATR

**DOI:** 10.1007/s00226-017-0967-9

**Published:** 2017-11-04

**Authors:** Mohamed Traoré, Joeri Kaal, Antonio Martínez Cortizas

**Affiliations:** 1 Ciencia do Sistema Terra, Departamento de Edafoloxía e Química Agrícola, Universidade de Santiago de Compostela, Campus Sur s/n, 15782 Santiago de Compostela, Spain; 2 Instituto de Ciencias del Patrimonio (Incipit), Consejo Superior de Investigaciones Científicas (CSIC), San Roque 2, 15704 Santiago de Compostela, Spain

## Abstract

Attenuated total reflectance-Fourier transform infrared (FTIR-ATR) spectroscopy was applied to 120 samples of heartwood rings from eight individual pine trees from different locations in Spain. *Pinus sylvestris* cores were collected at the Artikutza natural park (Ps-ART). *Pinus nigra* cores were collected in Sierra de Cazorla (Pn-LIN) and in La Sagra Mountain (Pn-LSA). Three discriminant analysis tests were performed using all bands (DF_T_), lignin bands only (DF_L_) and polysaccharides bands only (DF_P_), to explore the ability of FTIR-ATR to separate between species and growing location. The DF_L_ model enabled a good separation between pine species, whereas the DF_P_ model enabled differentiation for both species and growing location. The DF_T_ model enabled virtually perfect separation, based on two functions involving twelve FTIR bands. Discrimination between species was related to bands at 860 and 1655 cm^−1^, which were more intense in *P. sylvestris* samples, and bands at 1425 and 1635 cm^−1^, more intense in *P. nigra* samples. These vibrations were related to differences in lignin structure and polysaccharide linear chains. Discrimination between growing locations was mainly related to polysaccharide absorptions: at 900, 1085 and 1335 cm^−1^ more representative of Pn-LIN samples, and at 1105 and 1315 cm^−1^ mostly associated to Pn-LSA samples. These absorptions are related to β-glycosidic linkages (900 cm^−1^), cellulose and hemicellulose (C–O bonds, 1085 and 1105 cm^−1^) and content in amorphous/crystalline cellulose (1315 and 1335 cm^−1^). These results show that FTIR-ATR in combination with multivariate statistics can be a useful tool for species identification and provenancing for pine wood samples of unknown origin.

## Introduction

Wood is one of the most used materials for construction, furniture and other purposes (Holmgren et al. 1999; Tiscar and Linares 2011). It is a complex lignocellulosic material composed of cellulose, hemicellulose, lignin and extractives. Softwood from gymnosperms and hardwood from angiosperms are the major types of timber (Wing 2015). In the cross section of stems of many mature trees, two distinct regions can be distinguished, the sapwood (outer zone) and the heartwood (inner zone), the latter of which is usually relatively dark-coloured. The heartwood is composed of dead cells and usually has a darker appearance due to the abundance of extractive compounds such as tannin and terpenoids (Unger et al. 2001; Pandey 2005). The structural strength of heartwood enhances its durability, and heartwood is more resistant against environmental degradation processes such as irradiation and attack by fungi or other microorganisms as well (Taylor et al. 2002). Therefore, heartwood has been used rather than sapwood for most applications. In conifers such as *Pinus sp.*, the heartwood has a high occurrence of resin ducts, which distribute water and resinous substances. Oleoresin composed of volatile (mono- and sesquiterpenes) and non-volatile (diterpene: resin acid) resin is secreted by the duct to protect against insects and pathogens (Manninen et al. 2002).

During the modern age era, Iberian shipbuilders used pine timbers for planking and hulls (Castro 2008; Tiscar and Linares 2011), primarily from the dominant pine species in the Iberian Peninsula (Oliva et al. 2006; Tiscar and Linares 2011; Krakau et al. 2013), i.e. Scots pine (*Pinus sylvestris*) and black pine (*Pinus nigra*). These two types of pine occur preferentially on alkaline soils and have similar growth habitats (Reyes and Casal 2004). Even though these species can be easily differentiated according to morphological features related to their cones, needles and bark (Enescu et al. 2016; Houston Durrant et al. 2016), the species of archaeological timbers from the *Pinus* genus are not easily identified. To the authors` knowledge, there are no studies based on wood chemical composition to distinguish between these pine species. From previous studies on shipwreck materials, it was shown that infrared spectroscopy combined with statistical methods could be a useful tool for species differentiation (Traoré et al. 2016), which is very important regarding archaeological wood studies. The present work is the first attempt to deal with spectroscopic fingerprinting to distinguish between two *Pinus* species because of the ability of Fourier transform infrared (FTIR) spectroscopy to provide information on dominant functional groups present in wood samples.

FTIR is a powerful technique for wood characterization, providing details on functional groups and molecular bonds (Evans 1991; Pandey 1999; Popescu et al. 2007; Esteves et al. 2013), which are useful to identify wood parts, wood type and also assessment of wood quality (Moore and Owen 2001; Gandolfo et al. 2016). Due to the complexity of wood, most of the infrared bands cannot be directly assigned to a single component. Multivariate statistical techniques can be used to improve FTIR spectral analysis (Chen et al. 2010). For example, principal component analysis (PCA) has been applied to determine chemical differences between earlywood and latewood (Hori and Sugiyama 2003), or to distinguish between trees growing at different locations (Rana et al. 2008; Santoni et al. 2015). In a previous work, PCA was applied to transposed data matrices to study the effects of oxic and anoxic environmental conditions on archaeological wood composition (Traoré et al. 2016). Other multivariate statistics such as principal components regression (PCR), partial least squares (PLS) and discriminant analysis have also proven useful for translating variability in FTIR spectra to information on wood chemistry (Faix and Böttcher 1993; Bjarnestad and Dahlman 2002; Boeriu et al. 2004; Carballo-Meilán et al. 2016).

The aim of the present study is to differentiate between woods from two pine species (*P. sylvestris* and *P. nigra*) and trees growing at different locations in the Iberian Peninsula using FTIR-ATR in combination with stepwise discriminant analysis. The ability to distinguish between wood species and source area would be a valuable contribution to the discipline of provenancing archaeological timbers, and particularly to the assessment of the economy of Iberian shipbuilding wood of the XVI–XVII centuries (ForSEAdiscovery project, http://forseadiscovery.eu/).

## Materials and methods

### Description of samples

Eight individual trees of two pine species, *Pinus nigra* (black pine) and *Pinus sylvestris* (Scots pine) were studied, regarded as the most used ones for shipbuilding during the early modern era in southern Spain (Holmgren et al. 1999; Wing 2015).

**Fig. 1:**
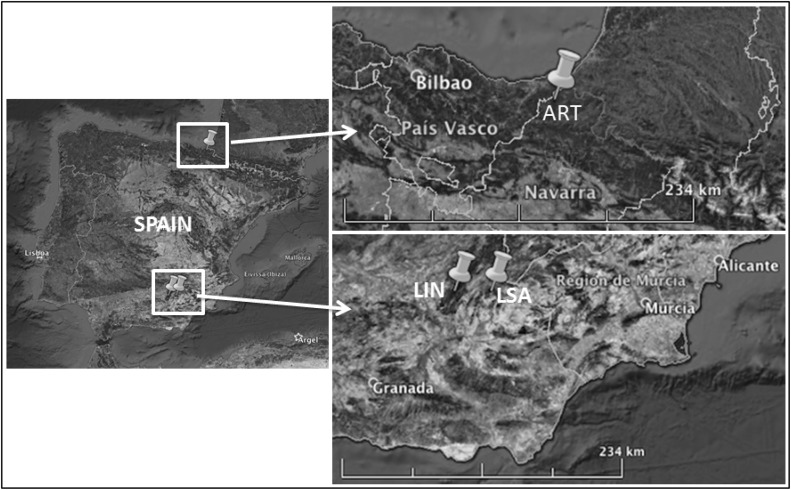
Map of the sampling locations (*LSA* La Sagra Mountain, *LIN* Linarejos, *ART* Artikutza)

In November 2014, six black pine trees were sampled in two different forests located in south-eastern Spain, in the region of Andalusia (Fig. 1). In Sierra de Cazorla the sampling was performed in the municipality of Linarejos (Pn-LIN), between 1100 and 1250 m above sea level (m a.s.l.). The forest in La Sagra Mountain (Pn-LSA) is located between 1800 and 2000 m a.s.l. Finally, two Scots pine trees were collected in May 2015 at the Artikutza (Ps-ART) natural park in the Basque Country (northern Spain, Fig. 1) between 450 and 500 m a.s.l.

The samples were collected from 100 to 150 years old trees. Wood cores were retrieved using an increment borer, at breast height and transported and stored in paper tubes. Prior to the analysis, the wood cores were oven dried at 30 °C for 2 weeks. Then, the surfaces were clean-cut on both sides to visualize tree ring patterns. Clearing the surfaces was necessary to improve the visibility of thin tree rings and to limit the effects of resin bleed.

Chemical treatment of wood samples was also performed to identify FTIR signals of resin, lignin and polysaccharides. For this purpose, 1.0 g of wood was ball-milled to powder and the extractives were obtained by extraction of 50 ml 1:1 toluene:EtOH solvent, followed by evaporation of the solvent. The residue was subjected to the classical 72% sulphuric acid treatment (“Klason lignin”), which eliminates polysaccharides. For details, refer to Rowell et al. (2005).

### Fourier transform infrared spectroscopy

For FTIR-ATR analyses, an Agilent Cary 630 FTIR Spectrometer equipped with a single-reflection diamond crystal was used. The spectra were collected in the absorbance range from 4000 to 400 cm^−1^ over 100 scans per sample, at a resolution of 4 cm^−1^.

Each wood core was cut ring by ring, and the FTIR measurements were taken on individual rings aiming to improve the contact between the sample and the diamond crystal of the FTIR equipment. However, for sections with very thin rings, several rings were analysed together. In this study, only data for the heartwood rings are presented and discussed, because this part is most commonly used as high quality timber, and because previous studies have shown that the differences in composition between sapwood and heartwood would otherwise dominate the variability (Traoré et al. 2016). For each tree, 15 spectra were recorded on randomly selected heartwood rings. In total, the data analysis was carried out on 120 spectra from 8 individual trees (see Table 1).

**Table 1:** Samples used in the present study

No.	Sites	Site codes	Tree ID	Species	No. recorded spectra
1	Artikutza	Ps-ART	Ps-ART-A	*P. sylvestris*	15
2	Artikutza	Ps-ART	Ps-ART-B	*P. sylvestris*	15
3	Linarejos (plot 1)	Pn-LIN01	Pn-LIN01-A	*P. nigra*	15
4	Linarejos (plot 1)	Pn-LIN01	Pn-LIN01-B	*P. nigra*	15
5	Linarejos (plot 3)	Pn-LIN03	Pn-LIN03-A	*P. nigra*	15
6	Linarejos (plot 3)	Pn-LIN03	Pn-LIN03-B	*P. nigra*	15
7	La Sagra	Pn-LSA	Pn-LSA-A	*P. nigra*	15
8	La Sagra	Pn-LSA	Pn-LSA-B	*P. nigra*	15
				Total	120

### Data analysis

Focus was put on the fingerprint region between 1800 and 800 cm^−1^ where most of the variations of the molecular bond vibrations occur (Pandey and Pitman 2003). In the untransformed absorbance spectra, many peaks are difficult to identify due to overlap. Therefore, the second derivative spectra were used for band selection using Resolutions Pro 5.3.0.0 of Agilent Technologies. The assignment of bands to specific molecular structures was based primarily on available literature (see Table 2). Principal component analysis (PCA) was also applied to the transposed data matrix to support the assignation of the infrared bands. This PCA allows for the identification of bands with similar variability, which is arguably indicative of a common precursor (López-Merino et al. 2012; Traoré et al. 2016). Moreover, FTIR was also performed on the individual wood constituents by isolation of extractives (“resin”) and the lignin residue after H_2_SO_4_ treatment (Klason lignin). The differential spectrum between the residue after resin extraction (lignin and polysaccharides) and the residue after acid digestion (lignin) is assumed to represent the polysaccharide fraction. Stepwise discriminant analysis was carried out on absorption data to determine the bands that allow differentiating between groups of samples. For the discriminant analyses, the spectra of each sample were used as input data, thus without grouping the 15 spectra of the individual trees. For the first discriminant analysis, two analyses were performed on lignin (DF_L_) and polysaccharide (DF_P_) bands separately, in order to identify the specific influence of these main wood components on the discrimination between samples. Secondly, all the selected bands were used in a global model (DF_T_), coding samples by species and forest location. For that purpose, from each individual tree 80% of the measurements (twelve spectra) were randomly used as training set (t) and the remaining 20% (three spectra) as validation set (v) to assess the accuracy of the discriminant models.

**Table 2:** Infrared bands and related molecular bond assignments

No.	Bands (cm^−1^)	Band assignments (References)	PCA factor^a^
1	805	Vibration of mannan in hemicellulose and CH out of plane bending in phenyl rings (Evans et al. 1992)	PC1
2	825	CH out of plane bending in guaiacyl units (Faix 1991)	PC3
3	860	C–H out of plane in position 2, 5, and 6 of guaiacyl units (Faix 1992; Boeriu et al. 2004)	PC3
4	900	CH deformation of beta-glycosidic linkages in cellulose (Evans et al. 1992; Faix and Böttcher 1992)	PC1
5	945	O–H out of plane deformation in carboxylic acid (Shearer 1989)	PC2
6	960	CH out of plane deformation in lignin (Popescu et al. 2007; Gandolfo et al. 2016)	PC3
7	985	CO stretching in cellulose (Herrera et al. 2014)	PC1
8	1005	C–O stretch in cellulose (Liang and Marchessault 1959)	PC1
9	1025	C–O stretching in primary alcohols in cellulose (Popescu et al. 2007)	PC1
10	1055	C–O stretching of secondary alcohols (Faix 1991)	PC1
11	1105	C–O–C stretching in cellulose and hemicellulose (McCann et al. 1992; Zhang et al. 2010)	PC1
12	1155	C–O–C asymmetric stretching in cellulose and hemicellulose (Faix and Böttcher 1992; Popescu et al. 2007)	PC1
13	1185	C–O stretching in Cellulose (Zhou et al. 2015)	PC1
14	1225	OH vibration in guaiacyl ring, C–C, C–O, and C=O stretches in lignin (Chen et al. 2010; Zhou et al. 2015)	–
15	1265	C–O vibration in guaiacyl rings (Popescu et al. 2007; Chen et al. 2010)	PC3
16	1315	CH_2_ wagging in crystalline cellulose (Colom and Carrillo 2005; Popescu et al. 2007)	PC1
17	1335	CH of methyl groups in methoxy in amorphous cellulose (Colom and Carrillo 2005; Popescu et al. 2007)	–
18	1360	C–H deformation in cellulose and hemicelluloses (Evans et al. 1992; Mohebby 2008)	PC1
19	1385	C–O stretching in cellulose and hemicellulose (Labbé et al. 2006)	–
20	1405	C=O in carboxylic groups in carboxylic acid, ester (Zhang et al. 2010)	PC2
21	1425	C–H asymmetric deformation in methoxyl, aromatic skeletal vibrations, lignin (Faix 1992; Popescu et al. 2007)	PC3
22	1465	C–H asymmetric deformation in methoxyl for lignins, asymmetric in—CH_3_ and CH_2_ in pyran for hemicellulose (Popescu et al. 2007; Chen et al. 2010)	PC3
23	1510	C=C stretching of the aromatic ring, C=O bond vibrations in extractive compounds (Popescu et al. 2007; Zhou et al. 2015)	PC3
24	1590	Skeletal vibrations from the C–C (Kubo and Kadla 2005; Vahur et al. 2011)	PC3
25	1610	C=O stretching conjugated to the aromatic ring, and in carboxylic groups in lignin, carboxylic acid, ester compounds (Zhao et al. 2014)	–
26	1635	Absorbed O–H and conjugated C–O in polysaccharides (Genest et al. 2013; Karunakaran et al. 2015)	–
27	1655	Absorbed O–H and conjugated C–O in polysaccharides (Genest et al. 2013; Karunakaran et al. 2015)	–
28	1690	C=O vibration in carboxylic group in resin acid (Mizzoni and Cesaro 2007; Vahur et al. 2011)	PC2
29	1730	C=O carbonyls in ester groups and acetyl group in xylan (Bodirlau and Teaca 2009; Zhou et al. 2015)	PC1

^a^Band assignment related to PCA factors (see Fig. 3: PC1 for polysaccharides, PC2 for terpenoids and PC3 for lignin)

One-way ANOVA was carried out to assess the significance of the differences between tree species and forest locations. The classification by homogenous subsets was done by the post hoc test of Student–Newman–Keuls, with alpha = 0.05. All statistical tests were done using SPSS 20.

## Results and discussion

### Absorbance and second derivative spectra

**Fig. 2:**
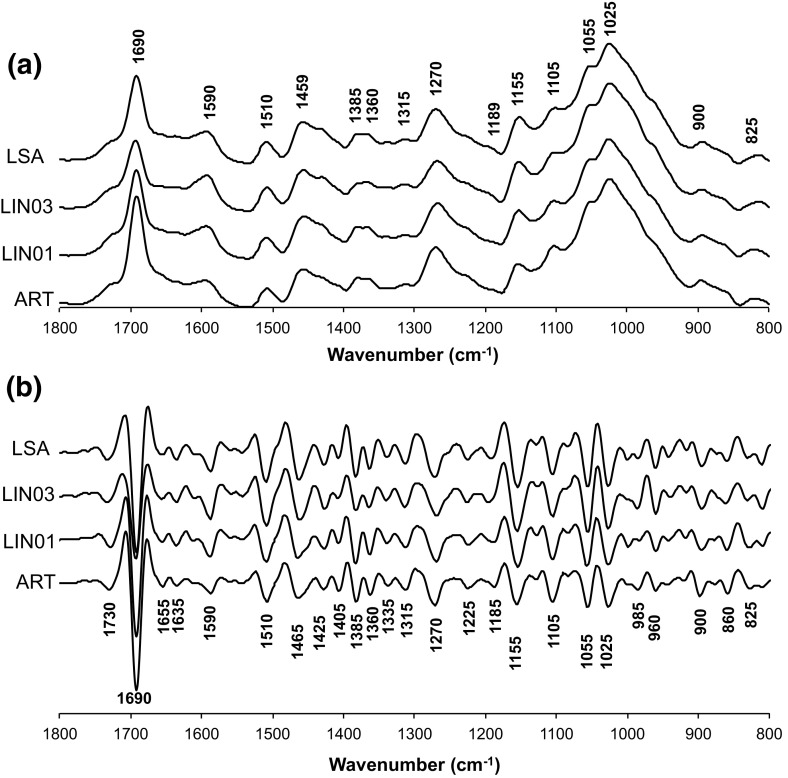
Average spectra (**a**) and second derivative spectra (**b**) of the heartwood samples (*LSA* La Sagra Mountain, *LIN01* and *LIN03* Linarejos plot 01 and 03, *ART* Artikutza)

Figure 2a shows the average FTIR spectra in the fingerprint region (between 1800 and 800 cm^−1^) of the pine heartwood samples. The strong absorbance at 1690 and 1025 cm^−1^, and moderate to weak absorbance at 1590, 1510, 1460, 1380, 1265, 1155, 1105, 900 and 825 cm^−1^, are associated with the major biopolymers of wood, i.e. cellulose, hemicellulose and lignin (see Table 2). Furthermore, the band at 1690 cm^−1^ is specific to resin acid compounds (Nuopponen et al. 2003; Vahur et al. 2011).

**Fig. 3:**
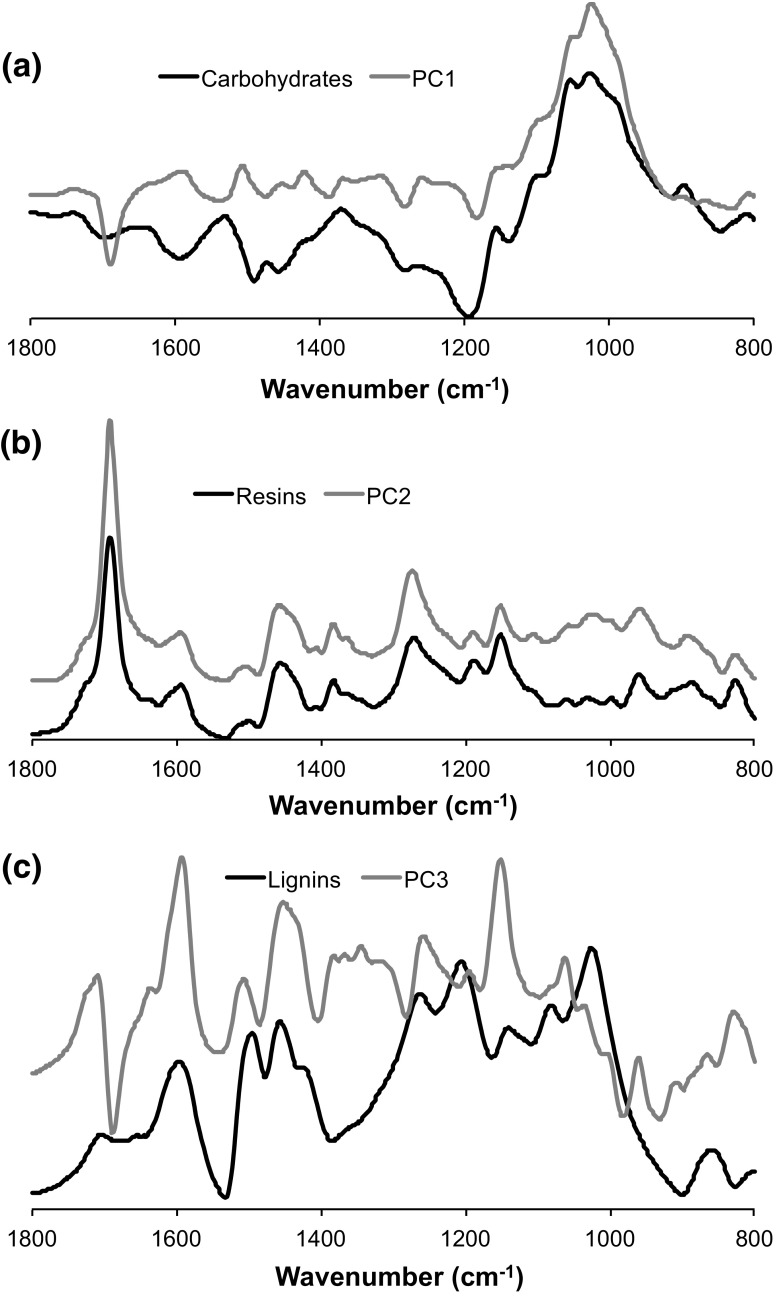
FTIR spectra of individual wood components (extracted resin, isolated lignin and carbohydrates from the difference spectrum of solvent-extracted wood and Klason lignin isolate), and the plot of component scores of the three first extracted factors from principal component analysis applied to the transposed data matrix (samples as variables)

In the second derivative spectra, many bands are identified more easily (Fig. 2b) confirming previous studies using the second derivative (Boeriu et al. 2004; Huang et al. 2008; Popescu et al. 2009; Zhang et al. 2016). The identified bands are listed with their most likely sources (based on literature) in Table 2. Most of the bands were assigned to lignin (825, 860, 960, 1225, 1265, 1425, 1465, 1510 and 1590 cm^−1^) and polysaccharides (805, 900, 985, 1005, 1025, 1055, 1105, 1155, 1185, 1315, 1335, 1360, 1385, 1635, 1655 and 1730 cm^−1^) (Faix 1991; Pandey 1999; Schwanninger et al. 2004). In general, the FTIR spectra of individual wood components (extractives, polysaccharides and lignin) support the band assignments given above. The FTIR spectrum of the extractives showed that it is composed almost exclusively of resinous materials (no bands from, e.g. tannin identified). Furthermore, the first three principal components from the PCA (accounting for 99% of the variation in the dataset) show striking resemblance with the spectra of the chemically isolated wood components (see Fig. 3 and also in Table 2). More specifically, PC1 is associated with polysaccharides (with higher scores for C–O bonds), PC2 with terpenoid constituents (high scores for carbonyl bonds) and PC3 with lignin (high scores for aromatic structural vibrations) (Fig. 3; Table 2). It is concluded that a combination of (1) comparison with band assignments from literature, (2) PCA of the whole dataset, and (3) chemical treatments for obtaining reference materials allowed for a very reliable identification of the primary structures responsible for the bands, including the minor ones, discussed in this study.

### Discriminant analysis

#### Discrimination based on lignin and polysaccharide absorption bands separately

**Fig. 4:**
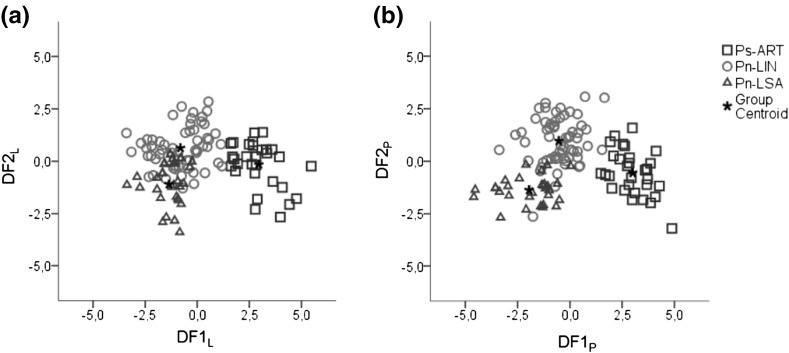
Plot of the canonical functions obtained in the discriminant analysis applied to lignin absorption bands (**a**) and polysaccharide absorption bands (**b**)

The stepwise discriminant analysis applied to the bands that were assigned to lignin (DF_L_) presented a clear separation (Fig. 4a) between *Pinus sylvestris* (Ps-ART) and *Pinus nigra* samples (Pn-LIN and Pn-LSA) on the first discriminant function (DF1_L_), which showed highest loadings for bands at 860, 1265, 1425, 1465 and 1510 cm^−1^. However, the distinction between the two *Pinus nigra* locations was poor. Using these four bands, the discriminant analysis was performed again coding only for species (*Pinus sylvestris* vs. *Pinus nigra*) in order to confirm the discrimination between them. The results showed a clear separation between Ps-ART samples, with positive scores, and Pn-LIN and Pn-LSA samples, with negative scores (data not shown).

The discriminant analysis on the bands related to polysaccharides (DF_P_) provided a good separation (Fig. 4b) between species on the first discriminant function (DF1_P_) and the two locations of *Pinus nigra* (Pn-LIN and Pn-LSA) were separated by the second discriminant function (DF2_P_). The associated bands are 805, 900, 1105, 1155, 1187, 1315, 1335, 1383, 1635 and 1655 cm^−1^. To confirm the discrimination related to forest location, another analysis was performed using only samples from *Pinus nigra* (Pn-LIN and Pn-LSA). Again, the results showed good separation, Pn-LIN samples showing positive and Pn-LSA samples negative scores.

#### Stepwise discriminant analysis with all absorption bands

**Fig. 5:**
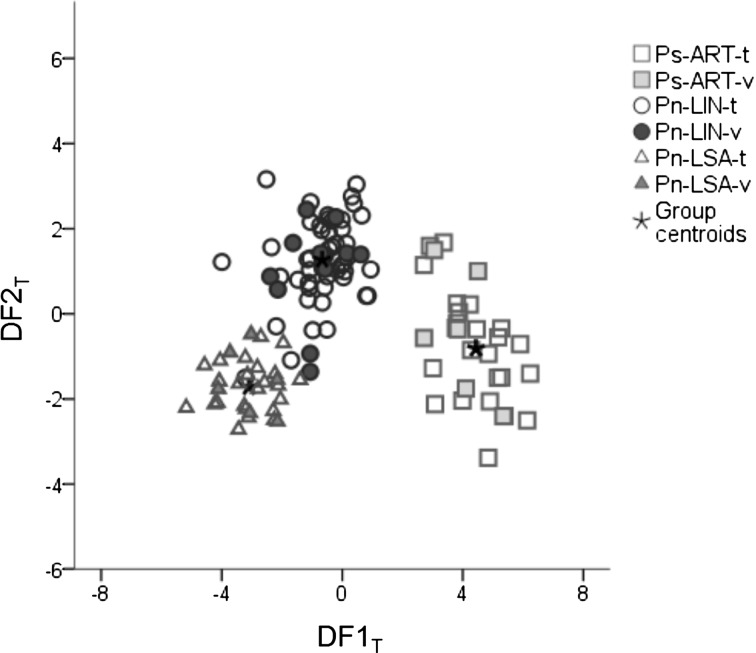
Plot of the canonical functions obtained by the discriminant analysis with all absorption bands (t for training set and v for validation set)

**Fig. 6:**
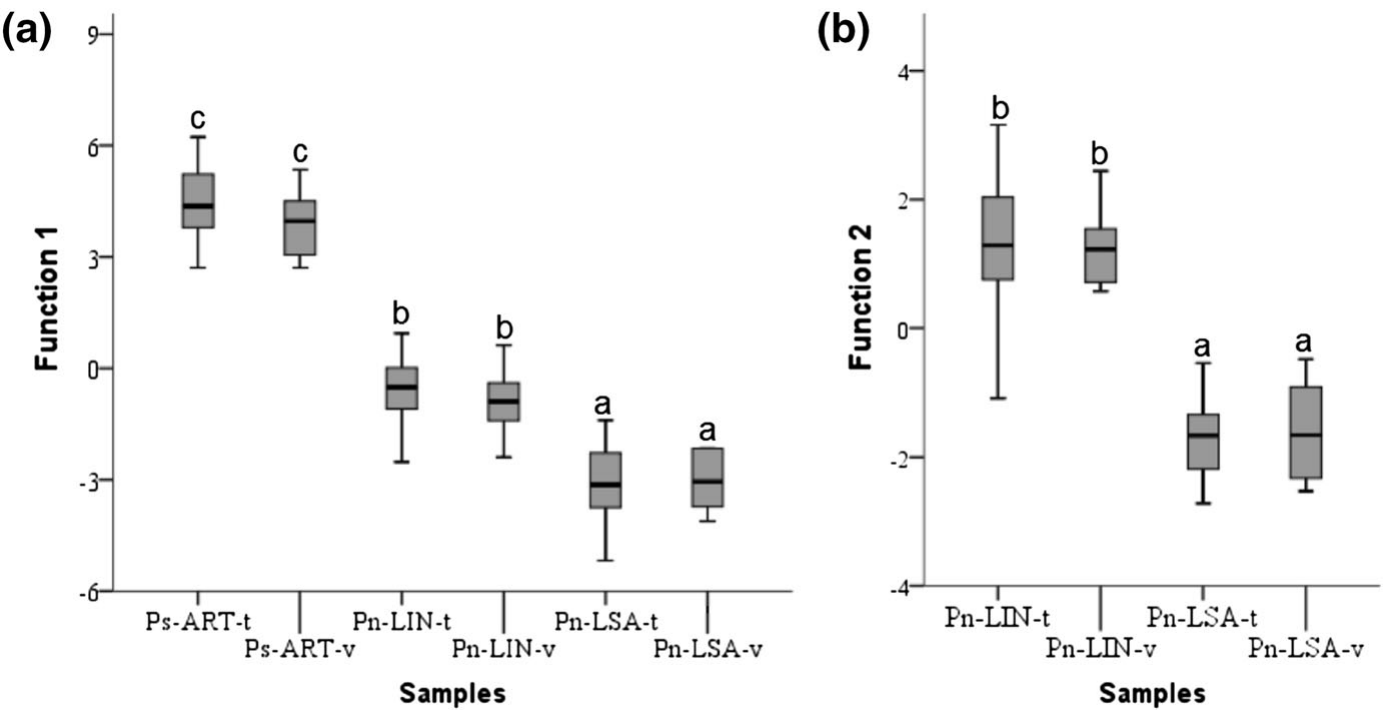
Boxplot of the discriminant scores of the training set (t) and validation set (v) displaying the accuracy of the discriminant model **a**: discriminant function 1; **b**: discriminant function 2. Groups are classified using Student–Newman–Keuls post hoc test (*P* < 0.001) in ascending order with label (*a*, *b*, and *c*)

The DF_T_ provided two discriminant functions that are based on twelve infrared bands (860, 900, 1105, 1225, 1315, 1335, 1385, 1405, 1425, 1610, 1635, 1655 cm^−1^). The first function (DF1_T_) explains 82% of the total variance while the second function (DF2_T_) accounts for the remaining 18%. The projection of the two functions (Fig. 5) shows that the samples plot in three main groups: Ps-ART, composed of samples of *Pinus sylvestris* trees from the Artikutza natural park; Pn-LIN, composed of samples of *Pinus nigra* trees from Linarejos, and Pn-LSA, composed of samples of *Pinus nigra* trees from La Sagra Mountain. Hence, DF1_T_ (0.94 canonical correlation) showed a clear separation between the samples of the training set of Ps-ART, with positive scores, and the samples of the training sets of Pn-LIN and Pn-LSA, which have predominantly negative scores. The ANOVA test indicated that this difference is significant (*P* < 0.001) for DF1_T_ scores (differences between pine species), as observed from boxplots (Fig. 6a). The DF2_T_ (0.80 canonical correlation) showed a clear separation between samples of the training sets of Pn-LIN and Pn-LSA, with positive scores for Pn-LIN and negative scores for Pn-LSA, albeit that there is some overlap between the clusters of Pn-LIN and Pn-LSA. A highly significant difference (*P* < 0.001) was also found for DF2_T_ scores (differences between locations for *Pinus nigra* trees) (Fig. 6b).

The validation set confirmed the accuracy of the discrimination between species and sampling locations. For the species differentiation (DF1_T_), *Pinus sylvestris* and *Pinus nigra* samples of the validation set were all correctly identified, with an average probability of 0.99 ± 0.01 (AVG ± SD); the same was found for each individual group of samples [Ps-ART-v (*n* = 6), Pn-LIN-v (*n* = 12) and Pn-LSA-v (*n* = 6)]. As for the growing location (DF2_T_), the samples of the validation sets of the two sites of *Pinus nigra* showed an average probability of correct site identification of 0.91 (SD 0.22). More specifically, samples from Pn-LIN-v were correctly assigned to their location with an average probability of 0.87 ± 0.26, whereas the probability for Pn-LSA-v was 0.99 ± 0.01. The lower probability for the Pn-LIN-v samples suggests that there is more variability in wood composition in the trees from this site. Even though it is known that the composition of arboreal wood depends on environmental factors of the growing location (Fritts 1976; Creber and Chaloner 1984), this variability can also be related to the sampling strategy (larger number of trees sampled). The relatively high chemical heterogeneity of the samples from Linarejos is also reflected by the relatively large distances of Pn-LIN samples to their group centroid in [DF1_T_-DF2_T_] space (Fig. 5). In fact, two samples of the training set and two of the validation set were grouped with Pn-LSA samples.

### Interpretation of absorption bands that discriminate between species and locations

To elucidate the chemical features of the wood that allow the models to differentiate between species and sites, the interpretation was limited to the lignin and polysaccharide bands that were identified from the individual discriminant models (DF_L_ and DF_P_) and by the global model (DF_T_), i.e. the bands at 860 and 1425 cm^−1^ for lignin and 900, 1105, 1315, 1335, 1385, 1635 and 1655 cm^−1^ for polysaccharides. The standardized canonical coefficients for the discriminant function are provided in Table 3. Bands with positive standardized canonical discriminant coefficients for DF1_T_ are more intense in Ps-ART samples (positive discriminant scores) and bands with negative coefficients are more intense in Pn-LIN and Pn-LSA samples (negative discriminant scores), whereas bands with positive coefficients for DF2_T_ show higher absorption in Pn-LIN samples (positive discriminant scores) and bands with negative coefficients show higher absorption in Pn-LSA samples (negative discriminant scores).

**Table 3:** Standardized canonical discriminant function coefficients for the DF_T_

Bands (cm^−1^)	DF1_T_	DF2_Total_
860	2.33	− 0.23
900	− 1.56	1.44
1105	1.62	1.08
1225	− 1.13	− 0.94
1315	− 1.83	− 3.83
1335	1.70	5.15
1385	2.21	1.11
1405	2.10	1.55
1425	− 3.44	− 0.94
1610	1.09	− 1.31
1635	− 4.28	0.95
1655	3.52	− 1.91

#### Lignin absorption bands

Firstly, the band at 860 cm^−1^ is associated with the CH out of plane bond vibrations in guaiacyl lignin (Evans 1991; Sills and Gossett 2012; Zhou et al. 2015). The positive standardized coefficient of this band implies that its absorbance is relatively strong in *Pinus sylvestris* wood. However, the band at 860 cm^−1^ can also be attributed to the propanoid side chain in lignin (Scheinmann 1970; Sharma 2004). Secondly, the band at 1425 cm^−1^ is assigned to the CH asymmetric deformation in methoxyl and aromatic skeletal vibrations in lignin (Faix 1992; Kubo and Kadla 2005). In this case, the standardized coefficients are negative, so that this band is associated with *Pinus nigra* samples. This suggests that aromatic methoxyl groups may be slightly more abundant in the structure of lignin in the *Pinus nigra* samples than the *Pinus sylvestris* samples.

These differences in lignin structure can be related to the lower shade tolerance of *Pinus sylvestris* than *Pinus nigra* (Trasobares et al. 2004). Lignin is the most receptive wood component to interactions with electromagnetic energy because of its macromolecular architecture (Chang et al. 1982). This higher sensitivity of *Pinus sylvestris* may be due to the lignin structure in the middle lamella, which is sensitive to sunlight exposure. Evans et al. (1992) showed that the CH out of plane bending vibration is the most influenced by factors that modify wood lignin composition. Thus, it is assumed that in *Pinus sylvestris* lignin structures with more CH out band bending vibration are produced in order to maintain phenylpropane derivatives, which are considered to protect meristem cells against light stress (Higuchi 1997).

The lignin signature was not useful for distinguishing different growing locations.

#### Polysaccharide absorption bands

For species differentiation, the band at 1635 cm^−1^ is relatively strong in *Pinus nigra,* whereas the band at 1655 cm^−1^ was associated with *Pinus sylvestris* samples. Both bands are related to OH bond vibrations including intermolecular hydrogen bonds between polysaccharide chains (Genest et al. 2013; Karunakaran et al. 2015). The intensity of hydrogen bonding exhibits strong influence on the rigidity of the cellulose chain and provides mechanical stability in fibres (Higuchi 1997; Klemm et al. 2004; Popescu et al. 2009). Karunakaran et al. (2015) stated that the band near 1635 cm^−1^ is characteristic of insoluble xylan. Insolubility could be due to the strong hydrogen bonds that lead to a large interaction between xylan chains (Poletto et al. 2014). Therefore, it is hypothesized that there are stronger interactions between xylan chains in *Pinus nigra* than in *Pinus sylvestris*. It had previously been found that xylan content could vary between wood species, even from the same genus (Timell 1964; Sjostrom 1981).

Regarding the differentiation of growing locations on the basis of polysaccharide FTIR signatures, the band near 900 cm^−1^ is stronger for pine samples from Linarejos (Pn-LIN) than for La Sagra Mountain (Pn-LSA). This band corresponds to the CH deformation of β-glycosidic linkages, which is related to the abundance of amorphous cellulose (Faix and Böttcher 1992; Evans et al. 1992). The bands at 1105 and 1385 cm^−1^ are assigned to the C–O stretching in cellulose and hemicellulose (Faix 1991; McCann et al. 1992; Zhang et al. 2010); however, the former, with a positive coefficient, seems to be associated with Pn-LIN samples while the latter, with a negative coefficient, seems associated with the Pn-LSA samples. There is no convincing explanation for this observation.

**Fig. 7:**
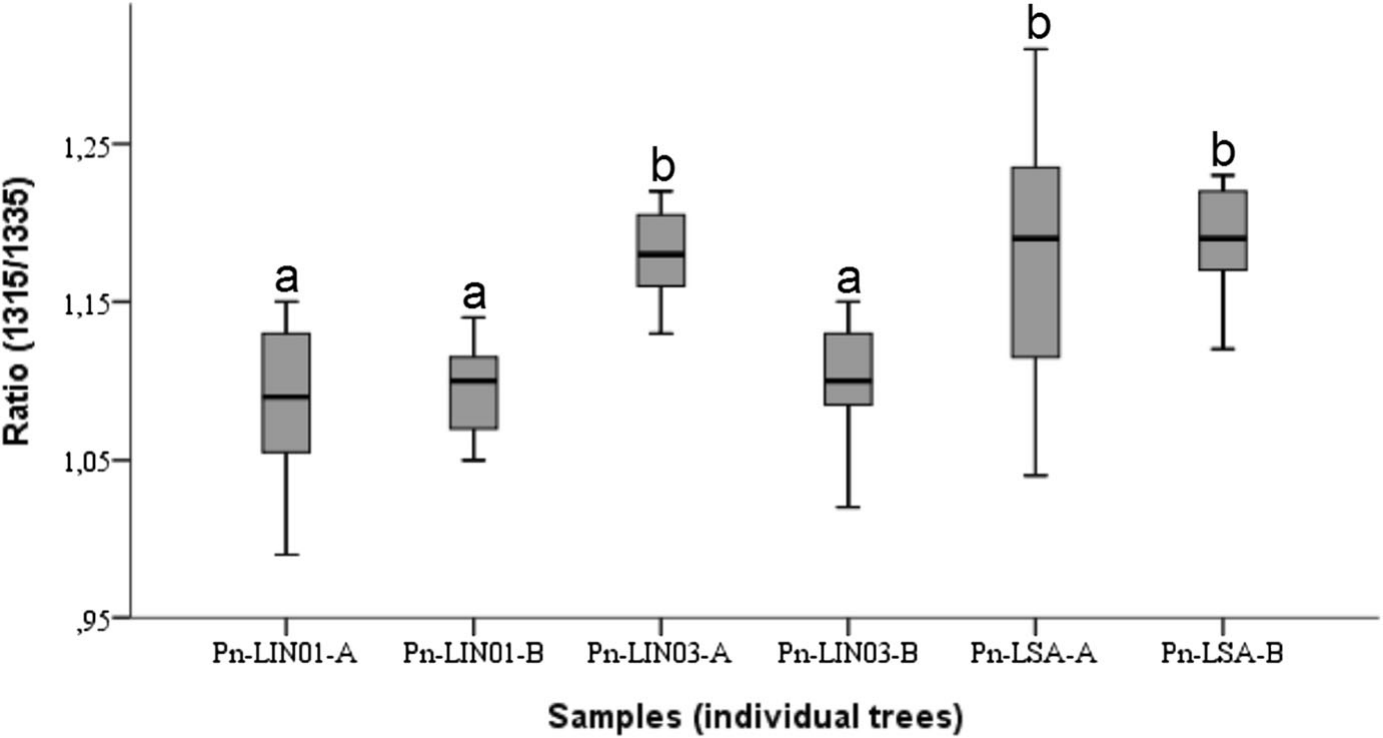
Boxplot of the ratio for amorphous-to-crystalline cellulose (1315/1335 cm^−1^) for *Pinus nigra* trees. Groups are classified using Student–Newman–Keuls post hoc test (*P* < 0.001) in ascending order (**a**, **b**)

The bands at 1335 and 1315 cm^−1^ are assigned to amorphous and crystalline cellulose, respectively (Colom and Carrillo 2005; Popescu et al. 2007). Samples from the Cazorla Mountain (Pn-LIN) had higher intensities of the band corresponding to the amorphous cellulosic structure, while samples from La Sagra Mountain (Pn-LSA) were characterized by higher intensities of the crystalline structure of cellulosic compounds. Indeed, the ratio between crystalline and amorphous cellulose bands (1315/1335 cm^−1^) indicates a relatively high degree of crystallinity in samples from LSA (Fig. 7). This ratio has been used as an empirical crystallinity index to compare amorphous and crystalline cellulose between hardwood and softwood (Colom et al. 2003; Colom and Carrillo 2005). The ANOVA test indicated that cellulose crystallinity is significantly higher (*P* < 0.001) in samples from La Sagra Mountain (Pn-LSA) than in those from Linarejos (Pn-LIN).

## Conclusion

It is concluded that the FTIR-ATR in combination with discriminant analysis allows to distinguish between the pine species studied (*Pinus nigra* and *Pinus sylvestris*) with high reliability, whereas the models also provide strong indications of geographic origin, at least for the two stands of *Pinus nigra* studied here. It appeared that the lignin composition can provide information on pine species, whereas the polysaccharide fingerprints and, in particular, its degree of crystallinity, are informative on both species and forest location. Even though the chemical background of the discriminant functions cannot be fully understood from this kind of analysis, these results show that FTIR fingerprinting is a useful tool for provenancing of extant wood. Despite this, a larger sample (i.e. more trees) would be recommendable in future studies to check for the consistency of the performance of the models. The current approach may also be applied to provenancing archaeological wood, in which the discriminating features are sufficiently well preserved. This will be further explored in upcoming studies.
